# Bridging the chronic care gap: HealthOne Mt Druitt, Australia

**DOI:** 10.5334/ijic.2243

**Published:** 2015-09-23

**Authors:** Justin McNab, James A. Gillespie

**Affiliations:** Menzies Centre for Health Policy, The University of Sydney, Sydney, Australia; Menzies Centre for Health Policy, The University of Sydney, Sydney, Australia

**Keywords:** HealthOne, chronic care, integration, care planning, community

## Abstract

HealthOne was part of a state-wide initiative to invest in new community-based facilities for collocating services. The HealthOne Mount Druitt is a virtual hub and spoke organisation established in 2006 in a socially disadvantaged part of Western Sydney based out of a new community health hub. The model is based on ‘virtual’ care planning and aims to improve coordination of care for older people with complex health needs, reduce unnecessary hospitalisations and ensure appropriate referral to community and specialist health services. General practitioner liaison nurses (GPLNs) work closely with clients as well as general practitioners (GPs) and other health care providers. Primary health care providers reported improved communication and coordination of services, and there have been lower levels of utilisation of the emergency department (ED) for patients following enrolment in the programme. HealthOne provides an example of how a virtual organisation together with highly skilled care coordinators can overcome some of the barriers to providing integrated care created by fragmented funding streams and care delivery systems.

## The intervention at a glance

### Organisation

HealthOne Mount Druitt is a virtual organisation based on a hub and spoke model of care. It is based in a socially disadvantaged area of Western Sydney, serving a culturally and linguistically diverse population. The programme is overseen by a Steering Committee which comprises general practitioners, Community Health, a branch of the Western Sydney Local Health District (WSLHD) (the authority which governs public hospitals in the region) and other service providers. Day to day management is provided by the Mount Druitt Community Health Centre which is owned by the New South Wales Ministry of Health.

### Objective

The programme aims to improve coordination and integration of services, and reduce unnecessary hospital readmissions, by improving referrals to specialised medical and other community health services for older people with complex needs.

### Target population

The target population are older people with chronic and complex illness who are at risk of further exacerbation and/ or hospitalisation and who would benefit from care planning and case management. To be enrolled in HealthOne the patients need to be eligible for complex, aged and chronic care services.

### Approach

Patients are referred to HealthOne by general practitioners, providers in the Western Sydney Local Health District, government and non-government organisations and family members. General practice liaison nurses organise multidisciplinary case conferences, coordinate care between various providers involved in the care of the patient, and ensure information is provided about the patient to the general practitioner or case manager. Patients and their carers and family members are invited to attend the case conference. A case manager is identified from among the multidisciplinary team and is not necessarily the general practitioner liaison nurse.

### Timeline

HealthOne Mount Druitt was established in 2006 and began enrolling chronic complex patients in September 2007. It was set up with initial funding from the NSW Treasury and Ministry of Health as part of a state-wide HealthOne programme. An independent evaluation of HealthOne Mount Druitt (HOMD) was completed in 2012.

### Results in brief

The vast majority of providers and all general practitioners involved agreed that HealthOne Mount Druitt had resulted in improved communication and information exchange between patients and providers and that the programme enhanced care coordination and improved the planning and coordination for patients. Patients admitted to the programme had fewer emergency department visits and shorter lengths of stay compared to the 12 months prior to enrolment. Referrals to allied health services rose but there were fewer referrals to less specialised community home nursing.

## Part 1: The model of integrated care

### Background

Any attempt at improving services integration in Australia must start by addressing a set of deep administrative and funding splits. Primary care has been the responsibility of the Commonwealth (federal) government, which funds general practitioners as gatekeepers for the health system. Medicare, the national health insurance system, pays rebates on fees for medical and a limited number of allied health services. In the case of general practice, general practitioners can currently elect to receive rebates directly from Medicare (‘bulk billing’), meaning the patient makes no payment. If the general practitioner wishes to charge more than the rebate set by Medicare, the patient pays an upfront fee and then has to collect the rebate from Medicare. Bulk billing covers the vast majority of services nationally (82% in December 2012) and is almost universal in poorer areas such as Mount Druitt, where few could afford a co-payment.

The massive gap between health outcomes in Aboriginal communities and the general population has led to a separate layer of health service provision. The Australian government funds the Aboriginal Medical Service Western Sydney, managed by local Aboriginal people for Aboriginal people, and a member of the National Aboriginal Community Controlled Health Organisation. Aboriginal Medical Service Western Sydney is located in Mount Druitt, near to HealthOne Mount Druitt and offers its patients integrated services, provided by salaried health workers, in contrast to the fee-for-service that dominants Medicare-funded primary care. HealthOne Mount Druitt refers Aboriginal patients into this parallel system where possible.

Public hospitals are funded and administered by state governments, with large indirect subsidies - but no direct administrative role - from the Commonwealth. All public health services are free, with no means test, but there may be a considerable waiting period for elective conditions. A large private hospital sector (around one third of bed days) is also subsidised by the Commonwealth, much of this indirectly through private health insurance. Private hospitals concentrate on elective surgery, while more complex procedures are largely in the public sector. This fragmentation has led to competitive relations between levels of government, with cost and blame shifting developed to high art forms [1].

Australia has experienced a pattern of constant reform of administrative arrangements in health care. Partisan differences over Medicare and universal health care have led to major funding changes with each change of government [2]. Reforms under the Rudd (2007–2010) and Gillard (2010–2013) Labour governments pushed states to decentralise the management of their public hospital systems, established stronger national reporting systems and a shift towards a standard national efficiency pricing (case mix) system. In primary care, the reforms established 61 regionally based Medicare Locals; primary health care organisations set up to coordinate primary health care delivery and tackle local health care needs and service gaps. Medicare Local boundaries match, as far as practicable, the boundaries set for the state hospital districts. Two central objectives have been the coordination of primary care services on a local basis: covering general practice, community pharmacy, allied health (physiotherapy, aged care, etc.) and Aboriginal health services. The Medicare Locals provide some primary health services to fill gaps - such as after hours - but not to compete with existing general practitioner practices. The area served by HealthOne Mount Druitt is covered by the Western Sydney Medicare Local, established July 2011. The election of a conservative coalition national government in September 2013 has increased the instability of the governance of primary care. The May 2014 federal Budget announced the abolition of Medicare Locals, which had been unpopular with sections of the Australian Medical Association. The shape of a possible successor is still unclear.

Improved management of chronic disease has been the subject of policy-making since the mid 1980s and has provided the main context for moves towards greater coordination of care [3,4]. The most commonly proposed solutions involve greater service integration, coordination, flexibility and continuity, along with policy and health system changes to improve the management of chronic disease [5,6].

The other national initiative at coordination came through changes in Medicare payments to provide incentives for chronic care planning. New item numbers were created for extended services, including an annual plan for patients with chronic illness, with referrals to a maximum of five appropriate services, such as physiotherapy, psychosocial services and podiatry. These incentives have been largely at the individual practitioner level and do little to overcome the institutional divide between primary and hospital care. At state level, coordination has come at hospital level, with resistance from general practitioners who, with some justice, have seen cost shifting as a major motivation.

### HealthOne

The HealthOne programme is a New South Wales (state government) health policy initiative that was first funded by NSW Treasury in 2005/2006. It built on a long history of chronic care planning and experimentation within the NSW Ministry of Health and at local level. It set out to integrate general practice and community health services with new models of multidisciplinary team care. Its goals included reducing avoidable hospital admissions, improving service access and health outcomes for disadvantaged and vulnerable groups, and building new sustainable models of health care delivery [7].

The HealthOne model broke from previous, failed coordination attempts which had been centred on hospital care. It was more flexible, allowing considerable local variation. Another successful early starter, the rural Cootamundra HealthOne, used a collocation of services model to integrate care. Urban services, such as HealthOne Mt Druitt, working across widely dispersed services in bigger population centres, were able to develop their own distinctive models of virtual integration.

**Figure 1.**
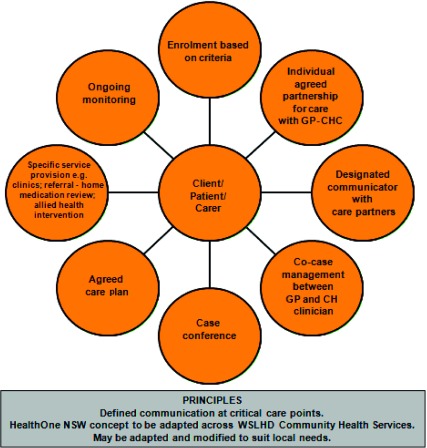
HealthOne Mt Druitt key service pathway elements.

HealthOne Mount Druitt was established in 2006 and began to enrol chronic and complex patients in September 2007. In 2008 patients began enrolling in a Child and Family services stream. The main goals of HealthOne Mount Druitt were to establish community-based models of care that were patient focused and flexible, as well as continuing, coordinated and comprehensive across the primary care–hospital interface. Care should be delivered in the most appropriate setting(s), e.g. home, general practitioner, other health facility, community outreach or community hub and patients should be active participants in care planning and management . Further, care should be provided by multidisciplinary teams (Figure 1) [8].

Mount Druitt was selected for the establishment of a HealthOne service because of its long-term high levels of socio-economic disadvantage and associated health needs. It is an outer metropolitan suburb, about 50 km west of Sydney’s CBD. Mount Druitt and its surrounding suburbs have a population of approximately 100,000, all within the larger Blacktown local government area. Mount Druitt scores highly in most measures of social disadvantage. About 38% of the population is living below the poverty line, and the area has lower education levels and higher levels of crime (especially juvenile) than metropolitan Sydney. Just under half the population live in public housing, compared to a Sydney average of 5.7%. Mount Druitt has higher unemployment, reported domestic violence and child protection notifications to social services than most of Sydney. Just over one-third of the population is foreign born, compared to a Sydney average of 23%, with large Aboriginal and Torres Strait Islander communities, who suffer considerably higher levels of disadvantage [9].

The area is well served with hospital services. Mount Druitt Hospital is a district public hospital linked to the nearby major metropolitan Blacktown Public Hospital. However, primary care services are fragmented and unevenly distributed. One hundred and thirty general practitioners work in the HealthOne Mt Druitt catchment area. About 71% work as sole practitioners, the remaining practices are small partnerships with a very few larger corporate entities. There are very few practice nurses employed, despite this being a growth area in Australian primary care.

Planning for HealthOne Mount Druitt was led by a Steering Committee with solid links into the local community through strong representation from local general practitioners and Community Health nursing services. The Steering Committee undertook a major study of local demographics and illness and service characteristics and consulted with general practitioners and Community Health staff members to identify three priority groups (chronic aged and complex care, children and their families and disadvantaged communities). Chronic aged and complex care was seen as the first priority, followed 12 months later by children and their families and then disadvantaged communities.

HealthOne Mount Druitt was the first and is still the most advanced HealthOne to be established and implemented in an urban setting. The programme is located within the larger Mount Druitt Community Health Centre in a purpose-built facility with approximately 120 multidisciplinary staff (nursing, therapy, counselling) with a part time spoke in the neighbouring suburb of Willmot, with a high Aboriginal population and limited health services. The programme also reaches people in their homes and at general practitioner practices through face-to-face visits and virtually by telephone (case conference, care and discharge planning).

Governance and oversight is provided by the HealthOne Mount Druitt Steering Committee, which includes the Community Health service, local general practitioners and representatives of the Medical Physicians Societies of Mount Druitt and Blacktown and the Western Sydney Medicare Local.

### Client group

HealthOne aims to meet the needs of:

- People with complex health needs or chronic illnesses living at home who have:
  ○ Multiple hospital admissions in a short period;
  ○ A complex condition that is challenging to manage;
- People who are frail and elderly and living at home;
- Pregnant women and families with young children where there are vulnerabilities/risk factors and a need for additional support (including children in Out of Home Care); and
- Parts of the local community that have difficulty accessing services due to cultural factors, disadvantage or isolation.

Eligibility criteria for enrolment are confirmed before entry into the programme and include at least one of the following: diagnosis of chronic and complex illness or acute exacerbation; severe end stage disease; risk factors for older age such as aged 75 plus years, risk of falls, cognitive impairment and reduced nutritional status, four or more emergency department/hospital presentations in the past 12 months and; readmission to hospital within 28 days. Other risk factors that can also be taken into consideration include lack of social support and the impacts of major life events. Of a sample of 125 HealthOne Mount Druitt patients enrolled in the chronic, aged and complex care service stream, the mean age was 68.4 years. The majority were female (58%). At least 24% of participants were born outside Australia.

A community health general and risk assessment tool is available for use by all community health staff external to the HealthOne Mount Druitt programme. Individual HealthOne Mount Druitt clients may have been assessed using this tool either prior to enrolment to HealthOne Mount Druitt or after entry into the programme. This assessment may be used by the programme but it is not required that all HealthOne Mount Druitt clients be assessed using this tool.

### Numbers of clients served by this programme

In the first few years of the programme, there were a total of almost 700 referrals, with a slight preponderance into the child and family programme. Ninety general practitioners were involved in the chronic and complex arm of the programme - either through referring patients or being contacted by the general practitioner liaison nurses (Table 1).

#### Approach to care

The linchpin of the HealthOne Mount Druitt model of care is the two general practitioner liaison nurses. Each patient is assigned to a general practitioner liaison nurse who identifies their needs based on previous assessments and their referral history. The general practitioner liaison nurses manage communications, case conferencing and care coordination between the various health professionals and other providers involved in the patient’s care. This may be done in person or by telephone (virtually) without the general practitioner or other service providers having to be physically present at the hub. Little use is currently made of more sophisticated communication systems, as many of the general practitioners have stand alone systems that do not integrate with other parts of the health system.

**Figure 2.**
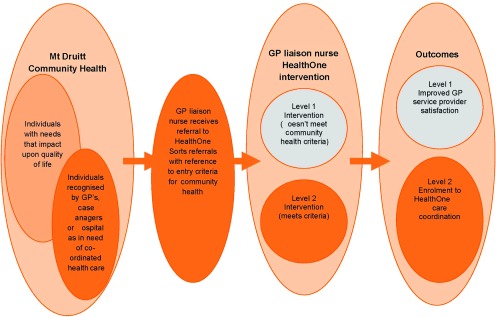
HealthOne complex, aged and chronic care model.

Patients’ needs are matched to the various components of the intervention through the programme’s case conferencing and care planning processes. Care planning may be initiated by the general practitioner liaison nurses, through a formal case conference or more informal exchanges of information. This information is then provided to the patient’s general practitioner or case manager and may be incorporated in a formal care plan. Patients, and their carers and family members if appropriate, are invited to attend the case conference where they participate in the discussion of care and treatment options with the multidisciplinary team (Figure 2).

Patient/family surveys are not routinely conducted to get feedback on satisfaction and experiences with care, although comments are invited on HealthOne Mount Druitt brochures. However, the recent evaluation of HealthOne Mount Druitt included an extensive qualitative study of patient and carer views on the programme. This research found that patients felt more supported and less anxious through their involvement with HealthOne Mount Druitt. Support for HealthOne Mount Druitt clients spanned from ‘just being there’ - a friendly ear (either on the telephone or by a home visit) - to practical assistance for medical and other health and social needs delivered through community health services or arranged by the general practitioner liaison nurse to be delivered through other health or social care providers. Patients also reported that community health staff, whether general practitioner liaison nurses or other community health staff, spoke to them in an open honest ‘truthful’ manner about their health and social situations.

Self management techniques and procedures, if considered appropriate for the patient and condition(s) in question, are discussed at case conferences where the patient is also present. Self care and management options may also be discussed with the patient at their general practitioner’s practice or in their home when visited by the general practitioner liaison nurse or other community or allied health staff.

### Management of coordination and care transitions

Overall responsibility for the management of care coordination rests with the HealthOne Mount Druitt general practitioner liaison nurse. Case management of individual patients is allocated by agreement within the multidisciplinary team on the most appropriate person to take on this role given the particular circumstances of the patient and their family.

On occasion, case conferencing can occur when the patient is in hospital and can include a discharge planner or hospital-based care coordinator to assist in the planning and management of the transition from hospital back to the patient’s home. The general practitioner is notified by the general practitioner liaison nurse 24–48 hours prior to a discharge teleconference so that they can participate if they wish.

Primary care doctors rely heavily on their relationship with the general practitioner liaison nurse. GPs involved with the programme report that the HealthOne Mount Druitt general practitioner liaison nurses provided a single point of contact; they were ‘user friendly’ and tailored their interactions to fit general practitioners’ needs. General practitioner liaison nurses organised care that the general practitioner could not through lack of time or because they did not have the knowledge of services available, e.g. Home Care, Counselling, other allied health services. General practitioner liaison nurses had the local knowledge of service boundaries and criteria for eligibility that general practitioners lacked. Aside from some referrals to public housing, HealthOne Mount Druitt made referrals into social care on a case by case basis. This is a common problem across Australian health and social services, which remain fragmented along departmental, state and federal and service delivery divides. The child and family arm of HealthOne Mount Druitt is more actively engaged with social care services, especially through school and other educational services-again, following a broader community pattern.

Electronic communications have remained relatively underdeveloped. General practitioners are most likely to communicate using fax, telephone and face-to-face visits, and increasingly by email, but with no use of telemedicine. It is through these communication processes that general practitioners are provided with information about their patients’ care and notified of changes in patients’ circumstances such as admissions to hospital or provision of other health or social services. General practitioners are involved in case conferencing and are provided with summaries of these discussions and other relevant information by the general practitioner liaison nurses in order to write care plans for their patients.

Currently, there is no electronic health record system that connects service providers across the HealthOne Mount Druitt catchment area. Some summary patient information is electronically available to both hospital (emergency department) and community health staff, including the HealthOne Mount Druitt general practitioner liaison nurses. Patients enrolled in HealthOne Mount Druitt are flagged by this system if they have presented to a hospital emergency department within the area or been admitted. The HealthOne Mount Druitt general practitioner liaison nurses can access this information and pass it on to general practitioners or other health service providers involved in patients’ care.

## Part 2: Implementation and organisation

The planning stage of HealthOne Mount Druitt took two years. The greatest challenge was building relationships between the key partners, especially overcoming strong established barriers to trust between general practitioners and Community Health.

The funding split between levels of government and forms of remuneration creates regular difficulties in the HealthOne Mount Druitt programme. Community health staff are employed on salary by the New South Wales state health department-funded Local Health District (LHD). The Local Health District manages public hospitals as well as community and population health services across the broader Western Sydney region. General practitioners work on fee-for-service under the Commonwealth Medicare system. General practitioners were paid for attendance at the Steering Committee but not for the numerous other tasks important to care coordination. Salaried staff had far more flexibility - a problem of time and resources that made general practitioner engagement a constant struggle. For general practitioners, time spent on care coordination reduced time for other patients.

These funding and administrative divides affected the readiness of the two main groups of health professionals to work together. Earlier failures at care integration shaped initial general practitioner perceptions of integration as an attempt to shift hospital problems and costs onto already overstretched primary care services. There was an established view from the Local Health District that failures were the result on an entrenched conservatism amongst general practitioners. Each view had enough of a basis to deepen the air of mutual suspicion.

HealthOne funding was initially limited to capital works. This was partly a NSW Treasury decision, but assumed that care coordination was primarily a matter of collocation of services. This was appropriate in rural services, but in the more scattered urban environment the emphasis on a ‘bricks and mortar’ hub was misplaced. While HealthOne Mount Druitt needed its physical location at Mount Druitt Community Health Centre, the real task lay in developing partnerships, processes and relationships amongst a fragmented workforce not inclined towards cooperation and deeply suspicious of initiatives identified with the state government. The planners of HealthOne Mount Druitt had to work around the restrictive framework set by the initial funding.

A weak local IT capability and the absence of an effective e-health record provided a barrier to the use of some of the more effective methods of integration/coordination, such as case conferencing using shared records. HealthOne Mount Druitt was forced to rely on available, time-consuming technologies: face-to-face partnership building work, telephone and paper communications, understood and used by the Mount Druitt general practitioners, leaving more sophisticated methods to the future.

Leadership proved a crucial ingredient needed to occur at a number of levels. The most important was organisational. HealthOne Mount Druitt needed to win the support, or at least tolerance, of local associations of general practice and from Community Health services within the Local Health District. It also needed sustained support at higher governmental levels (NSW Health and the regional Local Health District management) enabling policy change without attempting to micromanage local developments - a move that would have ended all chance of general practitioner participation. The strength of a small local leadership group of general practitioners committed to the HealthOne Mount Druitt project proved crucial. Their support was conditional on the state department and Local Health District management keeping its distance from the planning process, allowing a local alliance to develop between the general practitioner group and Community Health. Their initiatives and examples played a key role in overcoming mistrust and negative perceptions amongst their colleagues about the programme and the partners involved. Leaders from both the local general practitioner and Community Health groups had already won local respect and had a reputation for delivering good outcomes for patients and health professionals.

Community Health embraced new ways of working as a result of the implementation of HealthOne Mount Druitt and also changes within the Local Health District and the wider NSW health sector - including delivering services through two streams, chronic aged and complex care and child and family. Appreciating and respecting different roles, and being flexible in changing traditional organisational views, taking the time to be educated about ‘what general practice is’, assisted the change process for those involved with HealthOne Mount Druitt. The insight, vision or commitment of the programme partners in understanding that ‘things could be done better’ within the primary health care sector and that HealthOne Mount Druitt provided a mechanism through which improvements could be made also assisted in the change process. Community Health’s flexibility to allow individual workers to do what they needed to do ‘to make it work’ at organisational level, the local Community Health Centre and at the programme level (flexibility extended to how to work with general practitioners) also overcame mistrust and a previous history of perceived broken promises between general practitioners and the area hospital administration.

The diverse membership of the Steering Committee allowed partners to advocate for HealthOne Mount Druitt across the various levels of the health sector from NSW Health and Local Health District level to Community Health and local general practitioners allowing blocks to implementation to be overcome.

Care planning and case conferencing documents and other support from general practitioner liaison nurses allowed reimbursement of general practitioners under Medicare using fee-for-service reimbursement items that cover general practitioner management plans and team care arrangements. HealthOne Mount Druitt lightened general practitioners workloads by providing support to general practitioners who could then offer better care to their patients.

### Governance

The NSW Ministry of Health ‘owns’ HealthOne across all NSW sites, at least as a bricks and mortar affair. However, according to the rhetoric of partnership espoused within HealthOne Mount Druitt (sometimes referred to as ‘the philosophy of HealthOne’ by participants), all HealthOne Mount Druitt partners jointly own HealthOne Mount Druitt and so share responsibility and accountability for its implementation and smooth running.

The HealthOne Mount Druitt Steering Committee supplies strategic leadership and oversight. Day to day management is provided by the Mount Druitt Community Health Centre. This includes oversight of the division of care between the various partners, deciding who is responsible and accountable for providing which care under what circumstances. This responsibility does not go beyond coordination and the allocation of responsibility. The content of care remains a matter for each individual provider - general practitioners have their own professional regulation.

As the programme has grown in the Western Sydney region, with three other HealthOne sites, a new governing committee has been formed to manage common problems and coordination between sites. The new higher level of governance focuses on broader service planning across the Western Sydney Region, creating greater possibilities of integrating HealthOne primary care coordination more directly with hospitals, aged and social care. To this end, the Western Sydney Medicare Local has recently joined with the Western Sydney Local Health District and other partners to create a new senior management position dedicated to improving administration of integration across the sites.

In 2012, the NSW Ministry of Health was confident enough with the stability of the programme to commission a toolkit to standardise HealthOne policies, procedures, guidelines and administrative forms and processes based on the practices established at successful HealthOnes across the state. The toolkit will be used in the roll-out of HealthOne services in other locations throughout NSW.

### Resources - human and financial - committed to programme management

Since 2006/2007, the NSW Government has funded HealthOne NSW services to the tune of $46 million for capital development of integrated services across the state. About $3.3 million per annum of that has been made available to Local Health Districts for nursing, allied health and service integration positions. HealthOne Mount Druitt received $480,000 for the construction costs of the hub including a full fit out of clinical and office space as well as the leasing costs of its Willmot spoke.

The main costs of HealthOne Mount Druitt have been in salaries. Most of these are hard to calculate - general practitioners, especially those in leading roles, were paid a sitting fee to attend Steering Committee meetings, the Community Health staff on the liaison committee and other management functions are redeployed from other work within the Local Health District. The main distinct salary costs - purely devoted to HealthOne Mount Druitt - are the two general practitioner liaison nurses. They are funded out of the Community Health budget - but a point of tension has been the grading of these positions. Both were appointed a relatively high nursing classification. General practitioners and HealthOne Mount Druitt staff have reported that their level of autonomous judgement was an important element of the success of the programme. Since HealthOne Mount Druitt procedures have stabilised, there has been continual pressure from more centralised parts of community health management to downgrade these positions as a cost-saving move.

## Part 3: Impact and sustainability

### Evidence of impact

An evaluation of HealthOne Mount Druitt was completed by the Menzies Centre for Health Policy at the University of Sydney in 2012. The evaluation included quantitative and qualitative elements that have been incorporated in this case study [10].

A survey of health providers involved with HealthOne Mount Druitt found that almost all respondents (*n* = 54) either agreed (*n* = 37, 66%) or strongly agreed (*n* = 17, 30%) that the general practitioner liaison nurse role facilitated effective communication and information exchange between clients and service providers. All general practitioners surveyed either agreed (*n* = 18, 75%) or strongly agreed (*n* = 6, 25%) with this statement.

The majority of respondents (*n* = 47, 84%) also agreed (*n* = 33, 59%) or strongly agreed (*n* = 14, 25%) that the general practitioner liaison nurse role crossed traditional boundaries to enhance care coordination between clients and service providers. Almost all respondents (*n* = 44, 94%) agreed (*n* = 31, 66%) or strongly agreed (*n* = 13, 28%) that case conferences improved planning and coordination for HealthOne Mount Druitt clients/patients.

For people admitted to the programme with chronic and complex conditions, the number of emergency department presentations and length of stay in the emergency department fell significantly in the 12 months following enrolment in HealthOne Mount Druitt when compared to the 12 months prior to enrolment (Table 2). Almost 30% of participants had no hospital presentations after enrolment in the programme.

Health service utilisation analyses (Table 3) also found a significant change in pattern of service utilisation in the 12 months after enrolment. Referrals to allied health services such as physiotherapy, podiatry, occupational therapy, dietetics and psychosocial services rose but there were fewer referrals to less specialised community home nursing. This result suggests that process of care coordination through HealthOne Mount Druitt identified and remedied a range of previously undetected service needs.

It is clear that changes in patient outcomes and health service utilisation have occurred among people with chronic and complex conditions enrolled in the HealthOne Mount Druitt programme. However, it is less clear whether these changes are the result of the activities of HealthOne Mount Druitt, as the before–after study design, and the absence of a comparison group make attribution of causality difficult.

### Sustainability and spread

HealthOne Mount Druitt could not continue without the driving force of leadership from key people within the partnership because the current health system is not structured to work in an integrative collaborative manner. HealthOne Mount Druitt requires a wider culture change that would ensure practices and processes relating to working in an integrative multidisciplinary manner were embedded in partner organisations and established throughout the primary health care sector. At the moment, the programme depends heavily on leadership from the general practitioners and community health workers on the steering committee.

General practitioner liaison nurses are vital to almost all aspects of the implementation, operation and sustainability of HealthOne Mount Druitt. Permanent and ongoing general practitioner liaison nurse positions would significantly contribute to the sustainability of the programme and sufficient funding to increase general practitioner liaison nurse numbers and areas of specialisation would expand the programme, building cooperation across the different HealthOne sites in western Sydney and achieving some efficiencies of scale. The seniority and expertise of the individuals in the general practitioner liaison nurse roles are also critical. General practitioner liaison nurses have to command the respect not only of their Community Health colleagues, but of general practitioners, a much harder task, and they must be leaders and change agents.

Weak electronic health record systems have set barriers in communication between fragmented legacy systems in the hospital and community health systems and the poor use of e-health in general practice. Better communications should reduce some of the costs, especially time spent by the general practitioner liaison nurses in travelling for face-to-face meetings.

### Replication elsewhere

The NSW Ministry of Health has developed guidelines for the establishment of HealthOne in other parts of the state. A more detailed practical Toolkit is being prepared with active involvement from HealthOne Mount Druitt. This takes into account the major differences between the virtual centre, hub and spoke model of a dense urban centre such as Mount Druitt and rural and remote areas.

Two possible lessons for other countries - allowing for the unique circumstances of the Australia’s fragmented funding system - are the approaches to bridging quite antagonistic professional cultures and gaps between private and public providers. Attempts at top down management have failed dismally. The great achievement of HealthOne was to recognise these questions of trust and cooperation as the central issues for effective integration. However, this bottom-up approach has limits. The lack of effective electronic health records and precarious funding of some of the Community Health services, especially the general practitioner liaison nurses, suggest that there needs to be a more effective dialogue with central leadership - setting stronger frameworks that enable local initiatives to bear fruit.

## Part 4: Barriers and facilitators to effective implementation

### Systemic and contextual factors

In the complex environment of Australian health care, it is hard to point to any single element that made HealthOne Mount Druitt possible and sustained the experiment. National policies have had little direct impact - but the moves towards regionalization in recent health reforms have helped create a space in which local health professionals were able to devise their own partnerships and solutions to the problem of integrated care. At state level, work on the impact of chronic care on public hospitals - driven by fears of its impact on state hospital budgets - created a space for creative thinking about chronic care that moved beyond better management of hospital treatment to the need for better integration of hospital and community management of chronic illness. Federal interest in chronic care also legitimised this focus. Finally, little new money was directed into service delivery. As a result, those shaping new systems of integration had no choice but to work with existing services and their providers. Success depended on developing more nuanced methods of engaging and giving primacy to general practitioners. Hence, the development of HealthOne Mount Druitt was not a neat, linear process in which policies and frameworks initiated at national and regional levels were simply implemented at the local level.

HealthOne Mount Druitt has enabled a greater degree of integrated health/social care decision-making than is common in Australian chronic care. Case conferencing allows more informed and appropriate decision-making around integration and coordination of services, and it also allowed the participation of non-health service providers, including the provision of social care. However, the latter still remains an underdeveloped area - like much of Australian health and social care, divided into fragmented funding silos.

Financial resources have been a continuing problem - there were sufficient resources to build the hub and employ the two general practitioner liaison nurses for a set period but there was a general view from interviewees that the commitment from the state health department could have come earlier and been more substantial, particularly in ongoing/operating costs such as employing the general practitioner liaison nurses. Little funding was provided to help manage the difficult change management tasks involved in linking practitioners used to working in isolation and within Community Health. The service has relied on the goodwill and commitment of staff well beyond the normal call of duty.

### Organisational factors

These issues of shared mission, values and cultural alignment as well as strength of leadership have been discussed extensively in earlier sections. They have not cohered yet in strong common governance arrangements; except in the form of the steering committee’s representation from all players. As HealthOne Mount Druitt has moved from planning and implementation to more routinised operations, the fragility of its reliance on individual leadership and personal relationships may cause longer term problems. There are few shared organisational, clinical and financial facilities; although there are some activities at the hub, the ‘virtual’ component is where most of the activity occurs.

HealthOne Mount Druitt draws strength from being grounded in local community practitioners and organisations. It has a strong sense of accountability to the local community as well as the formal relationship to the Local Health District and the state Ministry of Health. The role of the Medical Local, only formed in 2012, is an unknown factor, but it has placed HealthOne as a central priority and can strengthen this connection to primary care providers and the community.

### Operational/service delivery factors

HealthOne Mount Druitt developed under adverse conditions - poor resources in a disadvantaged region of Sydney with fragmented systems with incentives pushing against effective integration.

Its success has depended on several organisational factors, including the established strengths of Community Health nurses in targeting and case finding and their experience supporting self-management through home visits. Multi-professional teamwork has developed slowly, but effectively, by demonstrating its advantages to often reluctant general practitioners through case conferencing and other informal interaction/information sharing in case planning. HealthOne Mount Druitt and its case conferencing/care planning has proved flexible in adjusting to patients’ changing needs - referring to more services or admittance to hospital if a patient needed more intensive care or organising appropriate support on a patient’s discharge from hospital.

It has also introduced a greater degree of patient and carer empowerment through the degree to which clients and/or families are expected to actively play part in care planning and implementation. Patients and families are there in the case conferencing if they wish and can participate to the extent that they want.

## Conclusions

The key lesson from the HealthOne Mount Druitt is that building trust and relationships between partners takes a long time and is fragile and easily dissipated if the favourable conditions created the supportive environment change - in this case, changing political and administrative and funding arrangements at regional, state and national levels have the potential to undo local achievements.

The most striking feature of HealthOne Mount Druitt has been its flexibility. The HealthOne framework has allowed the local organisation to identify particular needs and priorities and formulate models of care and appropriate organisational solutions that are not forced into a constricting template. This reflexive and experimental approach to leadership has been unusual in Australian health policy, but the basis for building effective local partnerships.

The work of the general practitioner liaison nurse, especially through informal and formal information sharing, and the case conferencing and care planning matching services (including, home and personal care) to peoples’ needs and context has enabled a model that goes well beyond the usual level of care in Australian general practice. Patients reported feeling a greater sense of security and being cared for, a ‘friendly ear’ or someone ‘just being there’. General practitioners also reported greater support and the unusual experience of being brought within the information loop of Community Health and other available services. This is a considerable improvement on a system in which under non-HealthOne Mount Druitt circumstances, a general practitioner may well not even know if a patient of theirs has been to hospital or has been receiving Community Health or other health and allied health services.

HealthOne Mount Druitt’s use of electronic communications is relatively undeveloped when compared with other developments in care integration in Australia. The current Western Sydney pilot of the national Personally Controlled Electronic Health Record offers some possibilities for innovation.

There will always be a need for a shared territory where general practitioners, Community Health and other service providers gather and plan for the future. At its simplest, perhaps the achievement of HealthOne Mount Druitt lies in the fact that health professionals in the primary health care sector within the Mount Druitt area have a sense of being connected rather than being isolated from ‘information loops’ and can better access information concerning patients and available service pathways, referral systems and eligibility criteria both within and outside the health sector.

The system could be improved. Greater resources - including funding to assist change management - would enable employment of more general practitioner liaison nurses and develop areas of specialisation targeting a broader range of priority groups. A further extension of the HealthOne Mount Druitt model could enable more sophisticated working relationships between general practitioner and Community Health with expanded multidisciplinary practice teams providing a greater range of outreach services.

Lessons learnt from developing and implementing the HealthOne Mount Druitt model could be used to inform, organisationally and structurally, a more integrated coordinated primary health care and acute care sector linked up to a suite of other health and non-health sector partners. However, this would require greater capacity and many more links, formal and informal, not only with health care providers but also with social and home care support agencies outside the health care sector, both non-government organisation and state run. This may extend to partnerships with the private sector. Considerably, greater resources for workforce and funding for further linkage programmes such as HealthOne Mount Druitt would be required.

## Figures and Tables

**Table 1.**
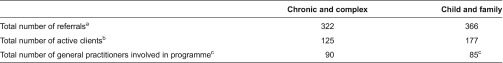
HealthOne Mount Druitt enrolments for chronic and complex/child and family, August 2011

Note: There is no current data on the duration of client time in the programme

^a^September 2007 to August 2011

^b^August 2011

^c^June 2011

**Table 2.**
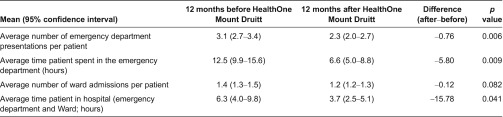
Average number of emergency department presentations, hospital admissions and time in the emergency department for 125 patients in the HealthOne Mt Druitt programme for 12 months before and after HealthOne Mount Druitt registration

Note: All values of mean and CI in Table 2 are back-transformed from the log-scale

**Table 3.**
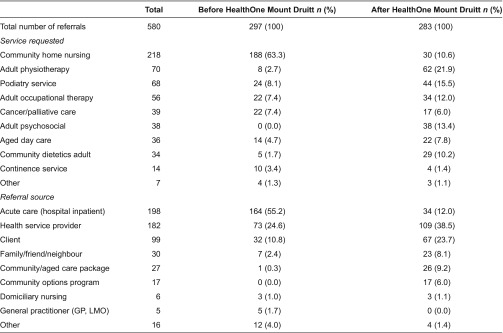
Number of referrals to community health services, with service type and referral source, among 125 HealthOne Mount Druitt patients in the 12 months before and after enrolment in HealthOne Mount Druitt

## NSW health planning documents

Background documents and policy frameworks that informed the development of NSW HealthOne services included: Improving Health Care for People with Chronic Illness: A Blueprint for Change 2001–2003; NSW Chronic Disease Prevention Strategy; Framework for Integrated Support and Management of Older People in the NSW Health Care System; NSW Chronic Care programme (Phase One 2000–2003, Phase Two 2003–2006, Phase Three 2006–2009), and most recently; Connecting Care. Relevant Community Health policy includes Integrated Primary and Community Health Policy 2007–2012 and Integrated Primary and Community Health Policy 2007–2012 Implementation Plan.
